# Modelling cost-effectiveness and value of information in clinical trials to inform stop go decisions: results from the arctic study

**DOI:** 10.1186/1745-6215-16-S2-O27

**Published:** 2015-11-16

**Authors:** Alison Smith, Peter Hall, John O'Dwyer, Claire Hulme, Dena Cohen, Walter Gregory

**Affiliations:** 1 University of Leeds, Yorkshire, UK; 2 University of Edinburgh, Edinburgh, UK

## Background

Trial interim analyses are traditionally based on an assessment of efficacy and safety. Early evaluation of cost-effectiveness and a quantification of the societal value of further research could provide additional information to inform stop-go decisions.

## Objective

To assess the potential utility of early cost-effectiveness analysis (CEA) and value of information analysis (VOIA) within the context of a randomised clinical trial.

## Methods

The ARCTIC trial randomised patients with previously untreated Chronic Lymphocytic Leukaemia to receive fludarabine, cyclophosphamide, mitoxantrone and low dose rituximab (FCM-miniR) or fludarabine, cyclophosphamide and rituximab (FCR; standard care). An interim efficacy analysis was conducted after 103 patients had completed therapy. CEA and VOIA were conducted using a Markov decision model, based on subsequent data from 200 patients.

## Results

The trial was terminated early based on the results of the interim efficacy analysis. FCM-MiniR was not expected to be cost-effective over a lifetime horizon, producing an average lifetime cost saving of £7,708 and health loss of -0.67 QALYs. The VOIA, however, suggested a high value of further research due to uncertainty around key parameters. Whilst the CEA results support the interim efficacy findings, the VOIA results highlight the cost of trial termination in terms of potential population net health loss (1,050 QALYs) by foregoing the opportunity to collect additional data.

## Conclusion

Early evaluation of cost-effectiveness within clinical trials could provide useful information in addition to efficacy data for interim analyses. Future research proposals should incorporate CEA and VOIA at interim analysis, allowing research-value to influence stop-go decisions.

